# Increasing Education Research Productivity: A Network Analysis

**DOI:** 10.5811/westjem.2019.12.44512

**Published:** 2019-12-19

**Authors:** William J. Peterson, Sally A. Santen, Joseph B. House, Laura R. Hopson, Meg Wolff, Michele Carney, John W. Cyrus

**Affiliations:** * University of Michigan, Department of Emergency Medicine, Ann Arbor, Michigan; † Virginia Commonwealth University, Department of Emergency Medicine, Richmond, Virginia; ‡ University of Michigan, Department of Emergency Medicine and Pediatrics, Ann Arbor, Michigan; § Virginia Commonwealth University, Virginia Commonwealth University Libraries, Richmond, Virginia

## Abstract

**Introduction:**

Forming effective networks is important for personal productivity and career development. Although critical for success, these networks are not well understood. The objective of this study was to usze a social network analysis tool to demonstrate the growth of institutional publication networks for education researchers and show how a single institution has expanded its publication network over time.

**Methods:**

Publications from a single institution’s medical education research group (MERG) were pulled since its inception in 2010 to 2019 using Web of Science to collect publication information. Using VOSViewer software, we formed and plotted a network sociogram comparing the first five years to the most recent 4.25 years to compare the institutions of authors from peer reviewed manuscripts published by this group.

**Results:**

We found 104 peer-reviewed research articles, editorials, abstracts, and reviews for the MERG authors between 2010 and 2019 involving 134 unique institutions. During 2010–2014, there were 26 publications involving 56 institutions. From 2015–2019, there were 78 publications involving 116 unique institutions.

**Conclusion:**

This brief report correlates successful research productivity in medical education with the presence of increased inter-institutional collaborations as demonstrated by network sociograms. Programs to intentionally expand collaborative networks may prove to be an important element of facilitating successful careers in medical education scholarship.

## INTRODUCTION

Education research and scholarship are important for dissemination of new educational practices and faculty promotion. As academic faculty, emergency medicine (EM) attendings are often charged to engage in the tripartite mission of clinical practice, education and scholarship, yet scholarship in medical education can be challenging.^1^,^2^ A better understanding of promotors of effective scholarly productivity will help the careers of EM academic faculty with a focus on medical education.

Research has elucidated several factors important for promoting scholarship including clear goal setting, a distinctive culture of research that emphasizes participation, frequent communication, accessible resources, and leadership with expertise and skill.^3^,^4^ One important component is the creation of an environment that facilitates productivity.^3^ Departmental educational research groups can facilitate and promote scholarship.^3^ Forming effective networks is an important part of personal productivity and career development and has positive effects on productivity of all individuals in a group.^3^,^5^ A network often begins within a department and extends well beyond the department, potentially leading to scholarly productivity. Although apparently helpful for success, these networks are not well understood.

Originating in the field of sociology, social network analysis (SNA) is a tool for analyzing the structure of connections between individuals or groups.^6^ SNA attempts to conceptualize a network using the ties (edges) that connect its members (nodes) and by focusing on attributes of the ties instead of the those of the members.^7^ This tool captures quantitative aspects of the patterns of relationships, which allows for quantitative comparisons between different groups and network structures. The application of SNA to the health sciences has become increasingly common as it is a useful tool for understanding connections within systems ranging from communication patterns between physicians to team functioning and structure.^8^–^10^ This has the advantage of showing connectedness that can reveal patterns. In addition, when compared over time, SNA can show growth of relationships between members of the network.

We hypothesize that social networks may contribute to successful education scholarship in EM. The objective of this study was to demonstrate the growth of institutional publication networks for education researchers in a medical education group, and use a network analysis tool to demonstrate this growth. We used a network analysis tool to show how a medical education research group (MERG) in a single institution expanded its publication network over time.

## METHODS

### Setting

The MERG was comprised of a group of faculty leaders from [blinded, single institution] emergency medicine (EM) residency, fellowship, and clerkship programs, as well as EM residents and fellows with a focus on education. The group intentionally formed as an innovative approach to promote educational work and turned usual educational work into scholarship by studying the impact of changes made to improve the programs. The scholarship was then presented at national meetings and often converted to a publication. The MERG team worked together, sharing projects that led to improved motivation, accountability, and work completion. The MERG had monthly meetings that served as brainstorming sessions for new projects, research skill building, and tracking work completion.^3^ These techniques led to a strong local network. As members developed their own expertise, they reached outside of the institution’s education group to national faculty to form broader networks for scholarship.

### Data Collection

We pulled all publications from each author of the University of Michigan MERG group using Web of Science (Clarivate Analytics, Philadelphia, PA, and London, GB). Web of Science is a subscription-based, inter-disciplinary database of scientific literature and conference abstracts that includes citations to the literature as well as information on how many times a specific item has been cited. The primary nine MERG faculty were used to generate the list of publications from the MERG group, and the author group was kept the same for the time period included in this analysis, 2010 to May 2019. Three authors were excluded as they were part of the initial MERG group but left education research shortly after MERG started to pursue other opportunities. Though the MERG group evolved to include other members during this time period, only the initial authors were included in this analysis to prevent the confounding of increased quantity of publications simply by expansion of members. Because we used publicly available data this study was considered to be not-human research.

### Analysis

**Figure 1:**
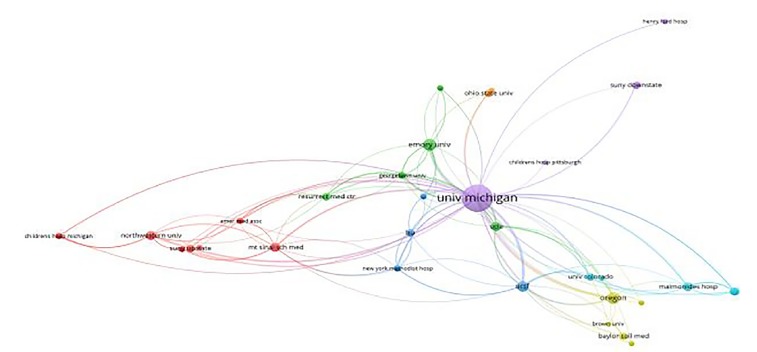
Institutions in 2010–2014. Points in the figures represent individual institutions (nodes), and lines (edges) between points represent the connections between institutions. Node size represents the overall number of times the institution was involved. The width of the connecting line represents the number of overall connections between institutions. The color represents clusters of closely related institutions measured by number of co-authorships within that range of years.

**Figure 2:**
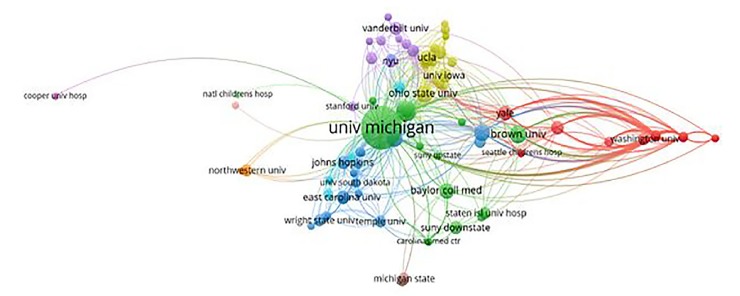
Institutions in 2015–2019. Points in the figures represent individual institutions (nodes), and lines (edges) between points represent the connections between institutions. Node size represents the overall number of times the institution was involved. The width of the connecting line represents the number of overall connections between institutions. The color represents clusters of closely related institutions measured by number of co-authorships within that range of years.

The publications as reported in Web of Science were recorded based on two time periods: the first five years 2010 to 2014, and the next 4.25 years from 2015 to 2019. This divide was chosen to compare two aggregates of time: the first five years, and the most recent five years. We recorded the number of publications, type of publications, and whether they extended beyond the local network. Using VOSviewer (www.vosviewer.com) we constructed a network sociogram based on the institutional affiliations of the author group involved in the publications from the first group, 2010–2014 (Figure 1), and compared it to the more recent group, 2015–2019 (Figure 2). Points in the figures represent individual institutions (nodes), and lines between points represent the connections between institutions.

The strength of connection, as seen in width of lines (edges) between nodes, was determined by analyzing co-authorship by organizations / institutions within the set of publications. In other words, the number of times two organizations were co-authors on a paper dictates the strength of connection. VOSviewer automatically calculates link strength based on co-authorship as part of the mapping process. The size of the node represents the overall number of times the institution was involved. The width of the connection line represents the number of overall connections between institutions. The color of the node represents clustering of nodes by the software to indicate institutions that are more closely related within the data set. In this case the clustering is indicative of the number of co-authorships with a range of years. Clusters are calculated by the VOSviewer software using an algorithm for mapping and clustering described more fully in Waltman, Van Eck, and Noyons.^11^

## RESULTS

**Figure 3:**
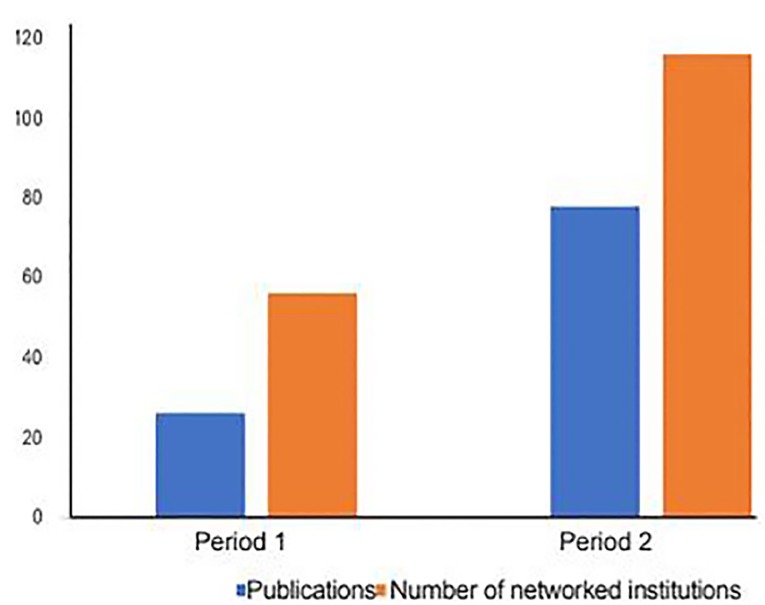
Publications for 2010–2014 (Period 1) and 2015–2019 (Period 2).

Using Web of Science, we found 104 peer-reviewed research articles, editorials, abstracts, and reviews for the MERG authors between 2010–2019. During 2010–2014, there were 26 publications (19 research articles and seven abstracts). Of these, 23 included authors from multiple institutions that included 56 unique institutions and three were from a single institution. From 2015–2019, there were 78 publications from the MERG author group (77 research articles and one abstract). Of these 58 included authors from other institutions and 20 were from a single institution. Over the time period 2010–2019, 134 unique institutions were involved in the co-authorship of publications from the MERG group. Fifty-six of these institutions were involved in publications over the 2010–2014 period, and 116 institutions were involved during the second period (Figure 3). The top 10 performing institutions for each year range by total link strength and number of items included in the analysis are reflected in Table 1.

The network sociogram illustrates the institutions involved in publications from the first years, 2010–2014 (Figure 1), compared to the most recent years, 2015–2019 (Figure 2). Points in the figures represent individual institutions (nodes), and lines (edges) between points represent the connections between institutions. Node size represents the overall number of times the institution was involved. The width of the connecting line represents the number of overall connections between institutions. The color represents clusters of closely related institutions organizations. In this study, institutions with the same color are closely connected subgroups via co-authorship. For example, in the 2010–2014 network, LSU is clustered with Boston University, New York Methodist, and UCSF but not Mt. Sinai. In this case, Mt. Sinai had co-authorship with LSU, but had stronger connections, through a greater number of co-authorships, with institutions in the red cluster. In the years 2010–2014 there were seven clusters with mean of eight institutions per cluster with a range of 2–15 in each cluster. In the years 2015–2019 there were 11 clusters with mean of 10.5 institutions per cluster with a range of 1–26 in each cluster.

The distance between nodes also represents the strength of connection between the nodes, meaning that nodes depicted as being further apart have weaker connections than those that are closer together or overlapping as in Figure 2. The two figures graphically illustrate evolving institutional relationships on a temporal basis as well as their relative strengths.

## DISCUSSION

The objective of this report was to demonstrate the evolution of the publication network for a research group at a single institution over time. The numbers of publications increased over time. In addition, as shown in the sociograms, the MERG network increased over time and evolved to include new institutions while prior relationships sometimes faded. While MERG has existed as a research group within one institution, the growth of the network over time has expanded to include co-authors from multiple institutions as demonstrated by comparison of Figure 1 to Figure 2.

These networks were facilitated by various learning networks such as service (committee work) and education involvement (didactics) seen in Table 2. Through a description of the different connection groups of the MERG network we hope to demonstrate how external networking can lead to increased scholarship. Some of the groups included clerkship directors academy (CDEM), residency education group (CORD), and pediatric EM fellowship program directors committee. Some faculty participated in MERC (Medical Education Research Certificate) at CORD; these connections resulted in multiple publications. In addition, some members expanded their work from exclusively EM-focused to general medical education with publications in high impact journals such as *Academic Medicine*. Some of the publications started as national meeting didactics and led to educational innovation reports, perspectives or educational monographs. Many of these groups continued to collaborate repeatedly for new scholarship.

## LIMITATIONS

There are several limitations to the study. One confounder is that two of the members of the group left [blinded institution] to work at another institution during the timeframe, and this likely accounts for some of the variation and expansion of the network. In addition, trainees left the institution and may be represented by their new institutions or came to our institution. In these cases, a perceived connection between institutions might not be considered to represent a new connection. However, the expansion of the research network extends beyond these known connections, and many of the new branches occur prior to those members moving to new institutions. An additional limitation is that some of the publication venues are not indexed in Web of Science, therefore some known publications are missing from this analysis.

## CONCLUSION

This brief report found associations between an increase research productivity in medical education with the presence of inter-institutional collaborations as demonstrated by network sociograms. Programs to intentionally expand collaborative networks, may be to be an important element of facilitating successful careers in medical education scholarship. Further investigation about successful research networks is needed.

## Figures and Tables

**Table 1:** Top 10 performing institutions for each year range by total link strength and number of items included in analysis.

2010–2014	Total link strength	Number of items	2015–2019	Total link strength	Number of items
University of Michigan	81	26	University of Michigan	237	78
UCSF	21	4	Brown	56	8
Emory	20	5	Harvard	54	7
Mount Sinai	19	3	University of Washington	48	7
Northwestern	18	3	Yale	48	5
Oregon	18	5	UCLA	42	6
UCLA	18	3	Columbia	40	4
Maimonides	14	3	VCU	40	12
Resurrection	14	2	Ohio State	36	7
LSU	12	2	East Carolina	30	4

*UCSF*, University of California San Francisco; *UCLA*, University of California Los Angeles; *LSU*, Louisiana State University; *VCU*, Virginia Commonwealth University.

**Table 2:** Network facilitators.

CDEM- Clerkship Directors in Emergency Medicine
MERC (Medical Education Research Certificate) at CORD
CORD- Council of Emergency Medicine Residency Directors and associated committees, task forces, and communities of practice.
University of Michigan Master in Health Professions Education (several MERG members were either enrolled in the program or mentors, leading to publications across institutions)
Didactic presentations at national and international meetings (Society for Academic Emergency Medicine (SAEM), Association of American Medical Colleges, Accreditation Council for Graduate Medical Education (ACGME), Council of Residency Directors, Association of Medical Educators in Europe (AMEE), Pediatric Academic Societies (PAS), American Academy of Pediatrics (AAP), Directors of Clinical Skills Courses (DOCs))
Standardized Video Interview developed by AAMC
American Academy of Pediatrics Section of Emergency Medicine Fellowship Directors Committee

*AAMC*, Association of American Medical Colleges.
